# Antibiotics as selectors and accelerators of diversity in the mechanisms of resistance: from the resistome to genetic plasticity in the β-lactamases world

**DOI:** 10.3389/fmicb.2013.00009

**Published:** 2013-02-08

**Authors:** Juan-Carlos Galán, Fernando González-Candelas, Jean-Marc Rolain, Rafael Cantón

**Affiliations:** ^1^Servicio de Microbiología, Hospital Universitario Ramón y CajalMadrid, Spain; ^2^Centros de Investigación Biomédica en Red en Epidemiología y Salud Pública, Instituto Ramón y Cajal de Investigación SanitariaMadrid, Spain; ^3^Unidad de Resistencia a Antibióticos y Virulencia Bacteriana Asociada al Consejo Superior de Investigaciones CientíficasMadrid, Spain; ^4^Joint Research Unit “Genomics and Health”, Centro Superior de Investigación en Salud Pública, Universitat de ValènciaBarcelona, Spain; ^5^Centros de Investigación Biomédica en Red en Epidemiología y Salud PúblicaBarcelona, Spain; ^6^Unité de Recherche sur les Maladies Infectieuses et Tropicales Emergents, Centre National de la Recherche Scientifique – Institut de Recherche pour le Développement, UMR 6236, Institut Hospitalo-Universitaire Méditerranée Infection, Aix-Marseille Université Faculté de Médecine et de PharmacieMarseille, France; ^7^Fédération de Microbiologie Clinique, Hôpital de la TimoneMarseille, France

**Keywords:** environmental resistome, intrinsic resistome, plasticity, β-lactamase

## Abstract

Antibiotics and antibiotic resistance determinants, natural molecules closely related to bacterial physiology and consistent with an ancient origin, are not only present in antibiotic-producing bacteria. Throughput sequencing technologies have revealed an unexpected reservoir of antibiotic resistance in the environment. These data suggest that co-evolution between antibiotic and antibiotic resistance genes has occurred since the beginning of time. This evolutionary race has probably been slow because of highly regulated processes and low antibiotic concentrations. Therefore to understand this global problem, a new variable must be introduced, that the antibiotic resistance is a natural event, inherent to life. However, the industrial production of natural and synthetic antibiotics has dramatically accelerated this race, selecting some of the many resistance genes present in nature and contributing to their diversification. One of the best models available to understand the biological impact of selection and diversification are β-lactamases. They constitute the most widespread mechanism of resistance, at least among pathogenic bacteria, with more than 1000 enzymes identified in the literature. In the last years, there has been growing concern about the description, spread, and diversification of β-lactamases with carbapenemase activity and AmpC-type in plasmids. Phylogenies of these enzymes help the understanding of the evolutionary forces driving their selection. Moreover, understanding the adaptive potential of β-lactamases contribute to exploration the evolutionary antagonists trajectories through the design of more efficient synthetic molecules. In this review, we attempt to analyze the antibiotic resistance problem from intrinsic and environmental resistomes to the adaptive potential of resistance genes and the driving forces involved in their diversification, in order to provide a global perspective of the resistance problem.

## INTRODUCTION

**FIGURE 1:**
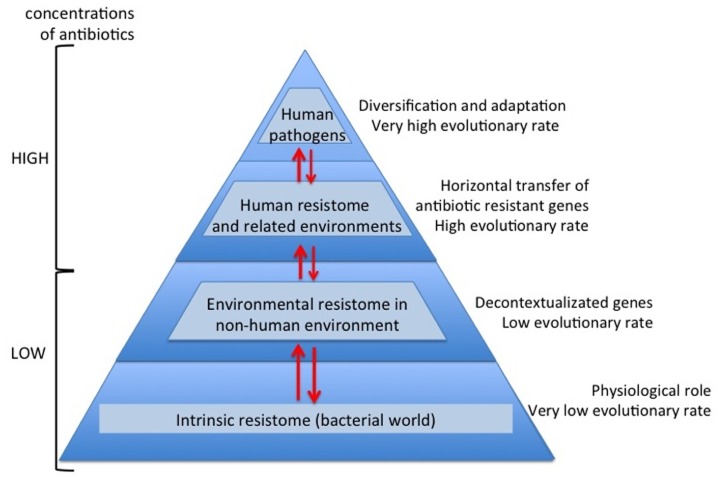
**Overview evolutionary processes of the antibiotic resistance**. During millions of years antibiotics and antibiotic resistance genes have co-evolved slowly. In this long period the first transition was the acquisition of pre-resistance genes by different bacteria (exaptation). This genetic transference allowed the evolution toward true and more efficient antibiotic resistance genes. However, the great evolutionary transition was the discovery, mass production and consumption of antibiotics. Antibiotics accelerated dramatically the diversification of resistance genes and selection for reaching extraordinary efficient variants.

Nowadays and thanks to the high-throughput sequencing tools and bioinformatics software, knowledge on high bacterial diversity in bacterial communities (metagenome) is increasing. A huge diversity of resistance mechanisms to practically all antibiotic families has been found in both antibiotic- and non-antibiotic-producing bacteria (D’Costa et al., 2006; Bhullar et al., 2012). Three types of resistome can be defined: intrinsic, environmental, and unknown (Fajardo et al., 2008; Martínez, 2008; Dantas and Sommer, 2012). In the intrinsic resistome or pre-resistome, the antibiotic resistant elements belong to bacterial metabolic networks, reflecting their role in microbial physiology. They might be coupled to signaling molecules (antibiotics) facilitating the co-selection of antibiotics and antibiotic resistance genes in a constant arms-race over a long time (Fajardo and Martínez, 2008). So today the intrinsic resistome is a wider concept and probably universal to the bacterial world. The description of the intrinsic resistome has expanded our knowledge about potential new resistance mechanisms (Dantas et al., 2008; Toleman et al., 2012). They could become true antibiotic resistance genes if appropriate driving forces were exerted. Moreover, the potential adaptiveness of these pre-resistome genes can be accelerated if, by chance, they are transferred to new genetic contexts (exaptation; **Figure 1**), where these genes may evolve toward more efficient enzymes without having a physiological role (Baquero et al., 2009; Gullberg et al., 2011). As a consequence, this silent and non-predictive resistome (unknown resistome) is ready to be selected (Baquero, 2012).

Efforts to understand the spread of antibiotic resistance and find previously undescribed antibiotic resistance mechanisms in non-human environments have yielded a more complex view of the antibiotic resistance problem (D’Costa et al., 2006; Dantas et al., 2008; Bhullar et al., 2012). Usually, the bacterial population-nutrient concentration ratio is low in the environment and a model of competitive interactions among microorganisms is established. Microbial competitors are able to produce high levels of natural antibiotics that become toxic compounds killing non-producer competitor strains in order to access the limited nutrients. However, antibiotic susceptible strains were able to develop strategies conferring resistance to these antibiotics, constituting the environmental resistome, starting adaptation, and co-adaptation cycles between antibiotics and antibiotic-resistant proteins (Martínez, 2009). New sequencing platforms have revealed that our old concept that only the antibiotic-producing bacteria were carriers of antibiotic resistance mechanisms was a very simple view. The scientific community has gradually begun to understand that antibiotic resistance has a global distribution in nature, even without the presence of humans, and that antibiotics and antibiotic resistance mechanisms have been evolving for millions of years (Wright, 2010).

The capture of antibiotic resistance or pre-resistance genes from intrinsic, environmental, or unknown resistomes is a stochastic, unpredictable phenomenon. Once the determinants of resistance reach the human environments or environments related to human activity, the evolutionary race between antibiotics and antibiotic resistance genes is drastically accelerated, as a consequence of the high concentration of antibiotics (Martínez et al., 2007). Therefore, antibiotics are not only selector agents of mechanisms of resistance; they are also accelerator agents of the evolution of resistance (**Figure 1**). This is a consequence of the huge plasticity observed in antibiotic resistance genes for acquiring a new spectrum of action or more efficient capacities with respect to the original spectrum (Deng et al., 2012). This observation suggests that the diversification force of resistance elements can be as fast and strong as the antibiotic diversification. For instance, the golden age in the commercialization for clinical use of β-lactam antibiotics was between the late 1970s and mid 1980s. Based on dated phylogenetic reconstructions of the most important β-lactamases in the clinical setting, the majority of diversification events occurred recently, even though the enzymes have been present for millions of years (Barlow and Hall, 2002a,b). *In vitro* models with CTX-M β-lactamases suggest that the explosive molecular diversification of these enzymes could only be explained by the simultaneous presence of different extended spectrum cephalosporins (Novais et al., 2010). Similar results were found with TEM enzymes (Salverda et al., 2011), suggesting that both the environmental contamination with different β-lactams and the potential plasticity of these enzymes could have been the main diversifying forces. Traditionally, the problem of antibiotic resistance has been focused as a clinical problem. Obviously, human health is the main reason, but we will never be able to cope with the antibiotic resistance problem if it is only seen as such. In this review, we attempt to analyze the antibiotic resistance problem from new perspectives. From the intrinsic resistome to the potential adaptiveness of determinants of resistance, from the environmental resistome to the driving forces involved in the diversity of variants related to specific determinants of resistance, as well as to provide a global view of the antibiotic resistance problem (Davies, 2007; Martínez et al., 2009).

## INTRINSIC RESISTANCE OR INTRINSIC RESISTOME

In the EUCAST expert rules on antimicrobial susceptibility testing, “intrinsic resistance” or “inherent resistance” is understood as a feature of all or almost all isolates of a bacterial species and in contrast to the acquired and/or mutational resistance concepts (Leclercq et al., 2011). From a microbiological point of view, intrinsic resistance can be a result of: (i) inherent difficulties for the antibiotic to reach its corresponding target due to impaired permeability of the bacterial envelope or efficient drug export systems, the so-called multi-drug resistance (MDR) efflux pumps; (ii) the absence of antimicrobial target(s) or presence of targets with low affinity; or even (iii) the presence of a mechanism that inhibits or destroys the antibiotics (enzymes that neutralize antibiotics in the cytoplasm or periplasmic space). Some examples of these mechanisms are included in **Table 1**. Nevertheless, all microorganisms contain efflux pumps involved in bacterial physiology, which can participate in resistance to different extents, although the clinical consequences might be of minor importance unless coupled with other resistance mechanisms (Li et al., 1994a,b; Piddock, 2006; Martínez, 2012; Nessar et al., 2012). Often, bacteria combine different mechanisms affecting several antimicrobial drugs. Conventionally, the intrinsic resistome has been defined as the set of chromosomal genes that are involved in intrinsic resistance and whose presence in strains of a bacterial species is independent of previous antibiotic exposure and is not due to horizontal gene transfer (Martínez, 2012).

**Table 1:** Different examples of intrinsic resistance with clinical relevance.

Intrinsic resistance mechanisms class	Resistance mechanism	Antibiotics affected	Microorganisms
Inadequate target	PBP5 mutations	Cephalosporins	*Enterococcus faecium*
Inactivating process	L1 beta-lactamase	Carbapenems	*Stenotrophomonas maltophilia*
Impaired permeability	Impermeable cell barrier	Vancomycin	Enterobacteriaceae

Nowadays, the “intrinsic resistome” is a wider concept than intrinsic resistance and includes genetic elements normally belonging to bacterial metabolic networks that can eventually participate in resistance to antimicrobial agents. Pre-resistance genes constituting the intrinsic resistome has two main characteristics: firstly, they confer low level of resistance which is also known as “pre-resistome” (Fajardo et al., 2008); secondly, the different resistance mechanisms are expressed at low levels, but their expression can increase due to the involvement of regulatory genes or as a consequence of mutational events, the so called “silent resistome” (Dantas and Sommer, 2012). Global transcriptome analyses of microorganisms grown in the presence of β-lactams induce the expression of *ampC* (Bagge et al., 2004). This chromosomal gene, present in the genome of *Pseudomonas aeruginosa* and of many Enterobacteriaceae for several hundred millions of years, plays a physiological role in the normal course of peptidoglycan synthesis, remodeling and recycling the bacterial envelope (Jacobs et al., 1994; Henderson et al., 1997).

For years recognition of the intrinsic resistome has not been an easy task. This will not be the case in the coming years as the implementation of whole genome sequencing strategies and bioinformatic tools for genome comparisons and knockout procedures will increase the recognition of determinants that might be involved in resistance phenotypes, even if they are very lowly expressed (Didelot et al., 2012; Huang et al., 2012; Schmieder and Edwards, 2012). The increased expression of these systems increases resistance levels (increased MIC values) whereas their absence increases bacterial susceptibility (decreased MIC values; Alekshun and Levy, 2007).

Hence, in bacterial resistance the differentiation between *intrinsic resistome* and *intrinsic resistance* is a thin line, but low-level resistance can be associated at a certain point to *intrinsic resistome* and high-level resistance to *intrinsic resistance* (Leclercq et al., 2011).

## ANTIBIOTICS AND ANTIBIOTIC RESISTANCE GENES HAVE ANCIENT ORIGINS

During the 1970s, it was shown that resistance genes related to aminoglycoside modifying enzymes (AMEs) in clinical bacteria had their origin in common soil bacteria belonging to *Actinomycetes*, which also produce AMEs (Benveniste and Davies, 1973). However, metagenome studies from different sources have questioned the “hypothesis of the antibiotic producers” because antibiotic resistance genes have been found in the microbiome of ancient isolated environments dating back a million years (D’Costa et al., 2006; Allen et al., 2010; Torres-Cortés et al., 2011; Bhullar et al., 2012). The functional role of antibiotic resistance genes in antibiotic-producing bacteria seems clear: they need to protect themselves from the activity of their own antimicrobials; however, in non-producers their role is less evident. Some explanations have been suggested. First, antibiotic resistance genes have or have had a physiological role in bacteria (see below). Second, antimicrobial agents are more prevalent than suspected. Analyses of bacterial genome sequences suggest that only 10% of the natural antibiotics have been discovered and probably only 1% of antimicrobial molecules from producers are known (Fischbach, 2009), possibly because they are natural products of bacterial physiology (Osbourn, 2010). More probably both scenarios are true and non-exclusionary. The complex network of physiological interactions that constitute the intrinsic resistome is coupled to small natural molecules, such as antibiotics, which might have a role as signaling molecules. According to this concept, antibiotics and antibiotic resistance genes have evolved during millions of years by forming interactions in coupled bacterial-specific networks, both by the same and different species (Arifuzzaman et al., 2006). For instance, different *Staphylococcus aureus* strains synthesize peptides that are recognized as signals by strains belonging to the same group and are competitive inhibitors against other *S. aureus* strains belonging to different lineages (Ji et al., 1997).

An accepted hypothesis is that these low molecular weight natural products (antibiotics) could originally be signaling molecules that help shaping the structure of microbial communities (Yim et al., 2007; Linares et al., 2006; Fajardo and Martínez, 2008). Even quinolone compound derivatives, which are produced by a variety of plants and microorganisms, have been found to act as quorum-sensing signal molecules, controlling the expression of virulence genes as a function of cell population density. This has been particularly investigated in *P. aeruginosa*. In this species, these compounds play multiple roles as membrane-interacting compounds, inhibitors of cytochrome complexes and iron chelators, as well as in the regulation of their biosynthesis and their integration into the intricate quorum-sensing regulatory networks governing virulence and secondary metabolite gene expression (Heeb et al., 2011). This could also be the case of other antibiotics, including small peptide molecules that might have a potential role in complex bacterial communities (microbiomes; Belda-Ferre et al., 2012). Therefore, the production of antibiotics is under strict genetic control. In resource-limited environments or when bacterial cells reach the stationary phase, the production of microbial secondary metabolites, such as microcins, is increased in order to yield a survival advantage to the producing bacteria (Romero et al., 2011). Microcins are DNA-damaging agents and, in consequence, the SOS regulon is induced and the chromosomal gene encoding the physiological inhibitor DNA gyrase, *gyrI*, is over-expressed (Baquero et al., 1995). This case is one of the examples showing that antibiotics and antibiotic resistance genes are natural products in constant and ancestral co-evolution (Chatterji and Nagaraja, 2002). Another interesting example is that of β-lactamases, which are structurally similar to PBPs (penicillin-binding proteins), the target of β-lactam antibiotics, the enzymes involved in the metabolism of peptidoglycan (Massova and Mobashery, 1998). Some PBPs such as PBP5 have weak β-lactamase activity conferring a low β-lactam resistance phenotype (Sarkar et al., 2011). In conclusion, antibiotics and also antibiotic resistance genes might have a dual functional and ecological role. At low concentrations antibiotics are signaling systems and at high concentrations they are weapons (Fajardo and Martínez, 2008).

## PHYSIOLOGICAL ROLE OF THE INTRINSIC RESISTOME ELEMENTS AND THE UNKNOWN OR SILENT RESISTOME

It has been anticipated that the intrinsic resistome elements have a physiological role in bacteria other than conferring resistance to antibiotics currently used in the clinical practice (Wright, 2007, 2010). A recent review addressing the importance of *Mycobacterium abscessus*, a rapidly growing bacteria involved in soft-tissue infections and chronic pulmonary diseases, showed the presence of a high number of resistance mechanisms responsible for natural resistance in this species, including efflux pumps, antibiotic-modifying enzymes, and target-modifying enzymes (Nessar et al., 2012). Most of these genes have physiological functions but express resistance in the presence of antibiotics, again suggesting that antibiotic resistance genes are the result of specific adaptive responses in genes with previous physiological roles.

The idea that intrinsic resistance is a consequence of the global bacterial physiology was later demonstrated with the study of the intrinsic resistomes of *P. aeruginosa* (Fajardo et al., 2008) and *Escherichia coli* (Tamae et al., 2008). *P. aeruginosa* is thought to be intrinsically resistant to several antimicrobial agents mainly due to a reduced cellular permeability and the activity of MDR efflux pumps. Fajardo et al. (2008) performed a transposon-tagging experiment to determine the elements involved in the intrinsic resistance of *P. aeruginosa*. They demonstrated that ~2% of the *P. aeruginosa* genome is involved in the altered susceptibility against different categories of antibiotics, but surprisingly only one gene had been annotated as an antibiotic resistance gene (Fajardo et al., 2008). Many of the genes detected as related to antibiotic resistance are involved in basic bacterial metabolism. However, their phenotypic effect is weak; therefore, the development of a real intrinsic resistance phenotype requires a complex assemblage of mutations, such as those previously commented in efflux pumps.

Those genes with a physiological function in the bacteria representing genes that do not confer clinical resistance to their native host, but are capable of expressing resistance when up-regulated or expressed in other hosts (exaptation) can eventually be involved in resistance will be part of the so-called “unknown resistome.” Elements from the intrinsic resistome and the unknown resistome have been stressed as potential targets for the design of new interventions to prevent antimicrobial resistance (Martínez, 2012).

## THE ENVIRONMENTAL RESISTOME

Although the origin of antibiotic resistance was mysterious in the past, it has become evident over the last decade that environmental bacteria were highly resistant to antibiotics (Wright, 2010). In fact this was discovered 40 years ago by Benveniste and Davies (1973) who found that AMEs from various species of the genus *Streptomyces* were similar to those found in clinical bacteria. Similarly, resistance to vancomycin, first reported in 1988 in *Enterococcus faecium* clinical isolates in Europe (Leclercq et al., 1988), was later found to be associated with glycopeptide antibiotic-producing *Actinomycetes* from soil (Marshall et al., 1997, 1998) as well as in non-antibiotic-producing bacteria of the genera *Paenibacillus* and *Rhodococcus* from soil (Guardabassi et al., 2004). More recently it has been demonstrated that several antibiotic resistance genes (genes conferring resistance to β-lactams, aminoglycosides, tetracyclines, sulfonamides, and phenicoles) have been exchanged between environmental bacteria and clinical pathogens on the basis of perfect nucleotide sequence identities of these genes with those from diverse human pathogens (Forsberg et al., 2012). In 2010, Knapp et al. (2010) quantitatively analyzed the abundance of 18 antibiotic resistance genes from five long-term-soil extracted DNAs from different areas in The Netherlands covering the years 1940 to 2008. They found that antibiotic resistance genes from all classes of antibiotics have increased since 1940, especially for tetracycline and β-lactam resistance encoding genes that have a higher abundance in the twenty-first century when compared with the 1970s, suggesting that levels of antibiotic resistance genes in the environment are now high and increasing (Knapp et al., 2010). Although resistant organisms in the environment may be the result of contamination by the recent use and misuse of antibiotics by humans, this previous dogma is now seriously disputed thanks again to the advances in new high throughput sequencing technologies (Rolain et al., 2012).

Aquatic environments are also a source for antibiotic resistance genes. The bacteria and/or antibiotic resistance genes could be transmitted directly or indirectly from these environments to humans (Zhang et al., 2009) as result of contamination by the recent use of antibiotics in agriculture and aquaculture selecting these intrinsic resistance genes (Baquero et al., 2008; Zhang et al., 2009; Wright, 2010). Many different antibiotic resistance genes from different classes of antibiotics (tetracyclines, aminoglycosides, macrolides, chloramphenicol, vancomycin, sulfonamides, and β-lactams) have been detected in different water environments worldwide using different molecular techniques as reviewed recently (Baquero et al., 2008; Zhang et al., 2009). The recent emergence and spread of carbapenemase encoding genes in Gram-negative bacteria seems to have originated from bacterial species in water environments (Lupo et al., 2012). For example, partial OXA-23 like carbapenemase sequences have been detected in groundwater samples from the Katmandu Valley of Nepal (Tanaka et al., 2012) that were 100% similar to those of sequences found in human head lice from Senegal (Kempf et al., 2012), suggesting that arthropods may also be a source of antibiotic resistance genes in human pathogens. Recently, MDR *E. coli* and *Klebsiella* spp. with extended spectrum β-lactamase (ESBLs) have been cultured from municipal wastewater treatment plant effluents in the Czech Republic (Dolejska et al., 2011). Similarly, several bacterial species including human pathogens carrying NDM-1, recently discovered (Yong et al., 2009) and spreading worldwide (Rolain et al., 2010), have been detected in tap and drinking water as well as drain and sewage in India (Walsh et al., 2011; Shahid et al., 2012) and in seepage water (rivers, lakes, and water pools in streets) from Vietnam (Isozumi et al., 2012). These data implicate contaminated aquatic environments, as a likely site for the exchange of antibiotic resistance genes between the clinic and the environment (Baquero et al., 2008).

Wild animals and pets, especially their gut microbiota, may also be a source for antibiotic resistance genes that come in contact with humans (Allen et al., 2010). Pets, cats and dogs in particular, may be a source for antibiotic resistance genes that could be transmitted to humans and the environment and *vice versa* (Guardabassi et al., 2004). It has been shown that resistance to antibiotics in bacteria recovered from pets has increased markedly over the last decade, especially the emergence of MDR *Acinetobacter baumannii*, *E. coli*, *Salmonella enterica*,* Staphylococcus intermedius*, or methicillin-resistant *S. aureus* that could be a risk for transmission to humans (Lloyd, 2007). Gilliver et al. (1999) reported more than 10 years ago that antibiotic resistance was highly prevalent even in wild rodents that were believed not to be exposed to antibiotics. Similarly, vancomycin-resistant enterococci carrying the *vanA* gene have been isolated from feces of wild mammals in the United Kingdom (Mallon et al., 2002), OXA-23 carbapenemase encoding gene in *Acinetobacter* species from the gut microbiota of cattle in France (Poirel et al., 2012), or MDR *E. coli* in European wild boars (Poeta et al., 2009; Literak et al., 2010), pigs, and rodents (Literak et al., 2009). Wild birds may also be a source for antibiotic resistance genes and their dissemination by migratory birds, as recently exemplified using functional metagenomics of a huge diversity of antibiotic resistance genes in fecal samples from a breeding colony of gulls in the United States (Martiny et al., 2011) or ESBL *E. coli* in great cormorans and mallards in Europe (Tausova et al., 2012). Finally, it has been recently shown that feces from urban pigeons in Brazil contain MDR enterococci (da Silva et al., 2012). Similarly, MDR enterococci including *Enterococcus faecalis* and *Enterococcus gallinarum* isolates resistant to vancomycin and MDR *E. coli* have been recovered from feces of feral pigeons in the Czech Republic (Radimersky et al., 2010), suggesting that pigeons should be considered a risk species that may contribute to the spread of resistance in the urban environment.

In recent years, new technological approaches, especially functional metagenomics, have led to the characterization and discovery of many unknown antibiotic resistance genes, the so-called resistome, i.e., all antibiotic resistance genes at a global scale in any bacteria (Wright, 2007). Functional metagenomic approaches have been developed and used to decipher the resistome in soil samples by Handelsman (2004) in 2004 that led to the discovery of new antibiotic resistance genes such as AMEs and β-lactamases. The first study of the extent of this resistome was reported by D’Costa et al. (2006) from bacteria isolated from soils showing that many environmental bacteria were MDR and that half of the antibiotic resistance genes discovered were unknown. Moreover, novel mechanisms of resistance involving the inactivation of telithromycin and daptomycin, two antibiotics approved in the last decade were discovered thanks to this approach (D’Costa et al., 2006). Later, Dantas et al. (2008) confirmed the presence of bacteria from soils intrinsically resistant to many different classes of antibiotics.

Nowadays, there is evidence that these antibiotic resistance genes have an ancient origin as demonstrated by the recent discovery using metagenomic analysis of genes conferring resistance to β-lactams, tetracyclines, and glycopeptides in permafrost sediments dating from 30,000 years ago (D’Costa et al., 2011). Similarly, MDR bacteria have been recently cultured from soil samples of an over 4 million year old cave in New Mexico (Bhullar et al., 2012), revealing an unexpected reservoir of antibiotic resistance genes in the environment. Although environmental reservoirs are well known as a possible source of antibiotic resistance genes detected in human pathogens, reports of antibiotic resistance genes from environmental bacteria with a high level of sequence similarity to those from human pathogens previously reported are limited. CTX-M-8 β-lactamase in environmental bacteria of the genus *Kluyvera* (Poirel et al., 2002) or *qnrA*-like genes from marine and freshwater bacteria of the genus *Shewanella* (Poirel et al., 2005) are examples of antibiotic resistance transferred from environmental bacteria to human pathogens. But, the environmental resistome is not only a huge reservoir of antibiotic resistance genes; it is also a generator of new mechanisms of resistance. The finding of a new bifunctional β-lactamase in a remote Alaskan soil sample (Allen et al., 2009) or the chimeric origin of the New Delhi metallo-β-lactamase NDM as the consequence of a recombination event between AME and β-lactamase (Toleman et al., 2012) are good examples of new antibiotic resistance arising in the environment.

In summary, antibiotic resistance is a natural and ancient phenomenon and its recent increase of antibiotic resistance among pathogenic bacteria has been the result of the ancient and recent mobilization of resistance genes from these environmental and animal reservoirs to human pathogens. Nevertheless, in spite of the efforts to clearly identify transfer events from environmental to pathogenic strains, only a few examples have been documented. The paucity of genetic evidence to date this transference is more likely the consequence of under sampling rather than that these exchanges have not occurred. Our knowledge of the environmental resistome remains low and future studies should be focused on discovery of these new antibiotic resistance genes for the better understanding of the magnitude of this phenomenon in environmental reservoirs and its potential impact in human pathogens. Another reason for the few cases documented so far could be related to the complex interplay between resistome genotypes and resistance phenotypes, which could be context-depend. Only some specific genetic combinations of resistance genes, plasmids, strains, and microbial community can be successful. Therefore, in the absence of experimental evidence the spectrum of action will be difficult to assess (Dantas and Sommer, 2012). The high prevalence and diversity of both antibiotic resistance genes and bacterial species in the environment make these ecosystems an opportunity to decipher the mechanisms of transfer and usage of these genes in bacteria living in a sympatric lifestyle which is a key component for bacterial evolution (Diene et al., 2012). We need to understand the magnitude of this phenomenon in the future in order to be able to define new strategies to face the problem of antibiotic resistance globally and not only from a human medical viewpoint.

## MOBILIZATION AND EXPRESSION OF INTRINSIC RESISTOME ELEMENTS

Comparisons of soil microbiota with clinical pathogens have provided evidence of an ancient and recent exchange of antibiotic resistance genes (β-lactams, aminoglycosides, amphenicols, sulfonamides, and tetracyclines) as well as the multiple mobilizations of sequences between these communities. They demonstrate not only evidence of lateral exchange but also a mechanism for the dissemination of antibiotic resistance (Forsberg et al., 2012). If the assumption that the intrinsic resistance has an ancient origin in environmental genes, then it can also be accepted that these genes are transmitted to human pathogenic bacteria. Admittedly these genes could be mobilized from their ancestors. Among these intrinsic resistance genes, β-lactamases genes (*bla* genes) are considered one of the best examples and are also discussed in this review.

As previously commented, amino acid sequence analysis of PBPs and β-lactamases point to a common origin of these proteins (Bush, 1997; Massova and Mobashery, 1998). Nevertheless, different ancestors have been recognized and different evolutionary trajectories might have occurred (Cantón, 2007). The analysis of β-lactamase phylogeny is not congruent with the phylogenetic history of its carriers, as horizontal gene transfer, frequently produced over time, might have interfered in the evolutionary process of β-lactamases. In some cases, such as class D (OXA type) enzymes, they are present both in Gram-positive and Gram-negative bacteria whereas Class C enzymes are only present in Gram-negative organisms (unlike *Mycobacterium smegmatis*), reflecting different evolutionary trajectories. Along these trajectories, β-lactamases have had to undergo structural alterations to become efficient as β-lactam-hydrolyzing enzymes, avoiding the interaction with peptidoglycan (or peptidoglycan precursors), which are the substrates of PBPs. It has been hypothesized that this gives an advantage to β-lactamase-producing bacteria, particularly in soil communities, where natural β-lactam-producing bacteria might be normally present (Bush, 1997).

One of the most studied β-lactamases is the Class A enzymes. Within this group the most widely distributed enzyme is TEM-1, which was first described in 1963 (Datta and Kontomichalou, 1965). The *bla* gene encoding this enzyme is carried in a transposable element (Tn3) that is widely present in plasmids of different incompatibility groups (Hedges and Jacob, 1974). Despite its widespread presence all over the world and in many different pathogenic bacteria, including among others all Enterobacteriaceae, *Haemophilus influenzae*, *Neisseria gonorrhoeae*, and *P. aeruginosa*, its ancestor remains undetermined. This is not the case of the CTX-M enzymes, which were recognized as plasmid-mediated enzymes hydrolyzing extended-spectrum cephalosporins (i.e., cefotaxime) in 1989 (Bauernfeind et al., 1990), from environmental bacteria of the genus *Kluyvera* (Poirel et al., 2002).

Nowadays, the CTX-M enzymes are widely disseminated all over the world within Enterobacteriaceae (Cantón and Coque, 2006). The corresponding genes, the *bla*_CTX-M,_ have been found in chromosomal location in different species of the genus *Kluyvera*, which is generally accepted as the origin of these genes (Cantón et al., 2012). In the *Kluyvera* species, cefotaxime resistance has low levels of expression. Mobilization by insertion sequences (IS), such as IS*Ecp1* or IS*BR1*, has resulted in higher expression of resistance in *E. coli*, *K. pneumoniae*, and other clinically relevant Enterobacteriaceae (Poirel et al., 2003; Lartigue et al., 2006; Rodriguez-Martinez et al., 2006).

Similar to Class A enzymes, the mobilization of *bla*_OXA_ genes has been estimated to have occurred at different times, the first one a hundred million years ago, whereas the last mobilization occurred very recently. This could have been the case of the carbapenem-hydrolyzing β-lactamase OXA-48 and its derivative OXA-181, which differs from the former by four amino acid substitutions, which have been related with *Shewanella xiamenensis* (Potron et al., 2011). This is an environmental species from marine and freshwaters that contains the *bla*_OXA-181_ gene. OXA-48-producing Enterobacteriaceae are increasingly recognized worldwide whereas the detection of OXA-181-producing isolates remains scarce (Poirel et al., 2012). Similarly to some of the *bla*_CTX-M_ genes, the *bla*_OXA-181_ gene was also preceded by its insertion in the element IS*Ecp1*, which might also have been responsible for its mobilization.

Interestingly, other species from *Shewanella* might also play an important role as the origin and reservoirs of resistance determinants. They have been found to harbor not only different β-lactamases genes but also resistance genes affecting other antibiotic classes such as fluoroquinolones (*qnrA* gene in *Shewanella algae*; Poirel et al., 2005; Rodriguez-Martinez et al., 2005). In the case of β-lactamases, *Shewanella oneidensis* and* Shewanella algae* harbor chromosomal class D β-lactamases genes (*bla*_OXA-54_ and *bla*_OXA-55_, respectively) and *Shewanella livingstonensis* and *Shewanella frigidimarina* harbor chromosomal metallo-β-lactamase genes (*bla*_SLB-1_ and *bla*_SFB-1_, respectively; Poirel et al., 2005). None of these genes has been found yet in plasmids but they could eventually be mobilized to pathogenic bacteria. Unlike these cases, the *bla*_OXA-23_ gene, whose progenitor has been found in *Acinetobacter radioresistens*, is nowadays widely spread in *A. baumannii* (Walsh, 2008).

All these examples illustrate how the intrinsic resistome can be mobilized from environmental bacteria and expressed at different levels in new hosts. However, it is surprising that despite the huge environmental and intrinsic resistome in environmental and commensal bacteria we can identify transmission events in only a few cases (**Table 2**; Davies, 1997; Davies and Davies, 2010), suggesting the existence of biochemical, metabolic, and genetic constrictions (Dantas and Sommer, 2012). When we are capable of understanding the “global resistome,” i.e., the environmental and intrinsic (including silent) resistome, then all the bacterial potential for fighting other bacteria will be known and only then will we be able to fight the antibiotic war on equal terms. Then, the following steps in our understanding of the resistance process will be to define and reveal the adaptive potential of those physiological genes that evolved toward resistance genes.

**Table 2:** Examples of resistance mechanisms in clinical isolates that evolved from natural functions in environmental bacteria.

Antimicrobial group	Mechanisms	Related natural protein	Natural reservoirs
Aminoglycosides	Acetylation Phosphorylation	Histone-acetylases Protein kinases	*Streptomyces*
Tetracyclines	Efflux (mar)	Major facilitator superfamily EF-Tu, EF-G	*Streptomyces*
Chloramphenicol	Acetylation Efflux (mar)	Acetylases Major facilitator superfamily EF-Tu, EF-G	*Streptomyces*
Macrolides	Target mutation	50S ribosomal subunit	*Streptomyces*
β-lactams (methicillin)	PBP2a	Homologous PBP2a	*Staphylococcus sciuri*
β-lactams (carbapenems)	OXA-48 inactivating enzyme	Proteins participating in peptidoglycan synthesis	*Shewanella xiamenensis*
	OXA-23 inactivating enzyme	Proteins participating in peptidoglycan synthesis	*Acinetobacter radioresistens*
Fluoroquinolones	Topoisomerase protection	Qnr-like protein	*Shewanella algae*

## PHYLOGENY AND EVOLUTION OF β-LACTAMASES

Antibiotic resistance is probably one of the best examples of “evolution in action.” The basic mechanisms of resistance appeared early in the evolutionary time-scale and evolved to counter the new defenses put forward by other bacterial species to compete for resources or survival against competitors and predators (Baquero and Blázquez, 1997; Salmond and Welch, 2008; Davies and Davies, 2010). It has been commented several times throughout this review that antibiotic resistance genes were ancient elements with a physiological role as components of complex networks. The decontextualization of these genes from their original background (exaptation) allowed a slightly faster evolutionary process toward a real antibiotic resistance *per se*. However, it is certain that since the adoption of antibiotic therapy to combat infectious diseases in the mid-twentieth century, the pace of this evolutionary race has accelerated tremendously. The most relevant player in this game is natural selection, which results from the advantage gained by a mutant organism that can overcome more efficiently the deleterious effects of antibiotics, and also by antibiotic-producers, which are able to find a new way of evading these defenses. In fact, this important role of natural selection has been demonstrated at different scales, from the observed changes in the prevalence of antibiotic resistance genes to the molecular level, by identification of those amino acid residues that are the main targets of selection (Novais et al., 2010).

Notwithstanding the relevance of natural selection, other evolutionary mechanisms are also important in the evolution of antibiotic resistance. Obviously, the most important chance involved in evolutionary success is related to horizontal gene transfer and the integration of antibiotic resistance gene in widespread plasmids and/or clones, but this aspect is not considered in this review. Other chance events such as mutations may play an important role in the evolutionary trajectories followed by antibiotic resistance strains. This has been experimentally shown to occur in the evolution of resistance to β-lactams on at least two occasions. Novais et al. (2010) showed that depending on the first mutation to appear in a few critical positions, resistance to cefotaxime and ceftazidime might follow-up to three alternative routes in CTX-M-type β-lactamases. A similar result was obtained after the *in vitro* evolution of TEM-1 β-lactamase (Salverda et al., 2011).

Apart from contingency, other stochastic processes may also be important for the evolution of antibiotic resistance. In their analysis of CTX-M experimental evolution, Novais et al. (2010) found that Darwinian selection alone could not explain the evolution of the CTX-M variants with the highest resistance to cefotaxime and ceftazidime because the fitness gradient along the different alternative routes always involved at least some steps of reduced fitness. The alternative possibility discussed by Novais et al. implies the action of genetic drift and selection in heterogeneous environments to cross these fitness valleys. In consequence, the action of natural selection does not guarantee, on its own, the evolution of the genotype with the highest fitness. Therefore, it would be of interest to extend these analyses to other antibiotic resistance genes and to evaluate the prevalence and relevance of natural selection and other evolutionary mechanisms in their recent and ancient evolution.

β-Lactams are the most widely used antibiotics in clinical practice over the world. Strong selective pressures upon them have resulted in a continuous increase of resistant isolates (Pitout and Laupland, 2008). In addition, they have also driven the diversification of known β-lactamases genes (Bush and Jacoby, 2010), in order to increase the spectrum of action, and facilitated the acquisition of new mechanisms from the environmental resistome, such as CTX-M β-lactamases (Poirel et al., 2002). Since the 1990s, the identification of new β-lactamase variants has increased dramatically (Davies and Davies, 2010), especially as a consequence of new class A and D β-lactamase variants (Bush and Jacoby, 2010). Moreover, the description of new β-lactamases with carbapenemase activity such as NDM and KPC (*K*lebsiella producing carbapenemase; Bonomo, 2011; Cornaglia et al., 2011), outbreaks of known β-lactamases such as OXA-48 (Dimou et al., 2012) and the spread of *ampC* in plasmids (D’Andrea et al., 2011) have contributed to the current state of alarm (Bush, 2010). We have analyzed, for the better understanding of the factors and processes underlying the recent spread of β-lactamases, the evolutionary dynamics of three β-lactamase families, i.e., ESBL-OXA, CYM, and IMP, representing enzymes belonging to classes D, C, and B, respectively. We decided to exclude the most prevalent (Class A), such as TEM and CTX-M, because they are well characterized (Salverda et al., 2010; Cantón et al., 2012) and their extent diversity is the consequence of recent evolution in response to the clinical use of β-lactam antibiotics (Weinreich et al., 2006; Novais et al., 2010). We have also excluded other worrying cases for public health, such as NDM and KPC, because the diversity of their known variants is still very low for a phylogenetic analysis.

**FIGURE 2:**
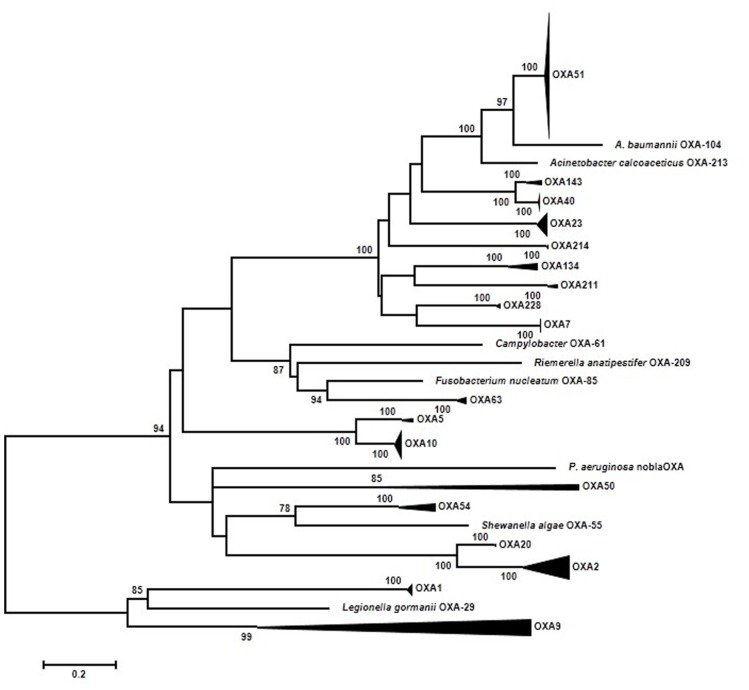
**Summarized representation of the maximum-likelihood tree of OXA genes obtained with the K2P + GI model of evolution**. Multiple nucleotide sequence alignments were obtained using the corresponding amino acid sequences with Muscle (Edgar, 2004a,b). Maximum-likelihood trees were obtained using the most appropriate model of evolution resulting from the comparison of Bayesian Information Criterion (BIC) values for 24 alternative models including gamma-distributed heterogeneous rates of evolution among and invariant sites. Support of the inferred clusters was evaluated with 1000 bootstrap replicates; BS values >70% are indicated. All these methods were used as implemented in MEGA 5 (Tamura et al., 2011). The scale bar corresponds to substitutions/site. The role of natural selection was investigated at the codon level by analyzing the difference between dN–dS substitution rates at different positions. Based on the corresponding maximum likelihood trees using a Muse–Gaut model (Muse and Gaut, 1994) of codon substitution and the Tamura–Nei model of nucleotide substitution (Tamura and Nei, 1993). Computation of dN/dS was performed with the Hyphy software package (Kosakovsky Pond et al., 2005). Additionally, two tests of neutrality were applied to the three data sets: the Fisher’s exact test of neutrality and a test based on bootstrapping (1000 replicates) of dN–dS values from which a variance of the corresponding statistic is derived in order to test the null hypothesis of neutrality (dN = dS). These tests were performed using MEGA 5 (Tamura et al., 2011).

It is already well understood that the OXA family is very diverse (Sanschagrin et al., 1995) and old (Barlow and Hall, 2002b). Only one phylogenetic analysis has been published using 35 OXA β-lactamases (Barlow and Hall, 2002b). The main conclusions of Barlow’s work were: first, four branches were identified (OXA-1/OXA-9; OXA-2; OXA-5; OXA-23); second, of the three mobilization events from a chromosomal site to plasmids detected two were ancestral events (OXA-1); and third, three branches (OXA-1, OXA-2, and OXA-10) were under significant positive selection at the beginning of the diversification process. A total of 192 ESBL-OXA sequences downloaded from GenBank were used in our new approach. In order to facilitate the interpretation of the resulting maximum-likelihood (ML) phylogenetic tree, highly supported (BS > 95%) clades of closely related sequences have been grouped (**Figure 2**). Each group has been denoted by a representative OXA variant present therein and the result essentially matches the nomenclature of similar groups in previous works (Couture et al., 1992; Poirel et al., 2010). Five major branches can be distinguished now. The new branch identified corresponds to OXA-61/63. The OXA-1/OXA-9 branch shows two different dynamics. The OXA-9 clade differs from other previous groups in its ancient origin, with long internal branches indicating long periods of evolution after divergence from the corresponding ancestors. On the contrary, the OXA-1 clade includes sequences from several different species such as *E. coli*, *P. aeruginosa*,* K. pneumoniae*, and others. They are very similar and suggest a very recent spread from a common ancestor. The OXA-51/OXA-23 branch includes 120 sequences, all but one from *Acinetobacter.* From these results, it is likely that this group of sequences originated in *Acinetobacter* and has remained distributed mostly within this genus. The only exception corresponds to a plasmid-borne representative isolated in *Klebsiella*, where it probably arrived from an *Acinetobacter* ancestor. The OXA-51 clade, the largest subgroup, is a very homogeneous group of sequences, with an average nucleotide distance of 0.018 substitutions/site (s/s) and a maximum within-group distance of 0.036 s/s. These results indicate that this group has diversified recently. Similarly, recent diversification events were suggested in the OXA-10 (the second largest group with 18 sequences) and OXA-2 clades. The average pairwise divergences were 0.022 s/s and 0.132 s/s and a maximum value of 0.045 s/s and 0.288 s/s, respectively. The OXA-54 clade, with very recently evolved sequences, was also found to be closely related to the OXA-2 clade.

These results are indicating that only some branches are evolving quickly and they could be under positive selection. Hence, the selective processes might have left an imprint in the form of increased rates of non-synonymous substitutions (dN) compared to those of synonymous substitutions (dS). In consequence, we have tested this hypothesis by comparing the rates of dS and dN substitutions at each codon. Based on the ML tree, estimates of the two substitution rates were obtained using the ancestral states inferred by ML under a Muse–Gaut model (Muse and Gaut, 1994) of codon substitution and the Tamura–Nei model of nucleotide substitution (Tamura and Nei, 1993). Computation of dN/dS was performed with the Hyphy software package (Kosakovsky Pond et al., 2005). Positions with <95% sequence coverage (i.e., with >5% of the sequences presenting a gap) were removed from the analysis. In contrast to the initial expectation, no codon presented a statistically significant higher rate of dN than dS substitutions. The same general results were obtained when the different groups in the OXA phylogenetic tree were analyzed separately in search of positively selected codons as well as in an analysis with the seven ungrouped sequences of this gene (**Figure 1**). This result indicates that neither the ancient divergence nor the recent spread of some groups of OXA alleles have been driven primarily by selection acting at this (codon) level.

**FIGURE 3:**
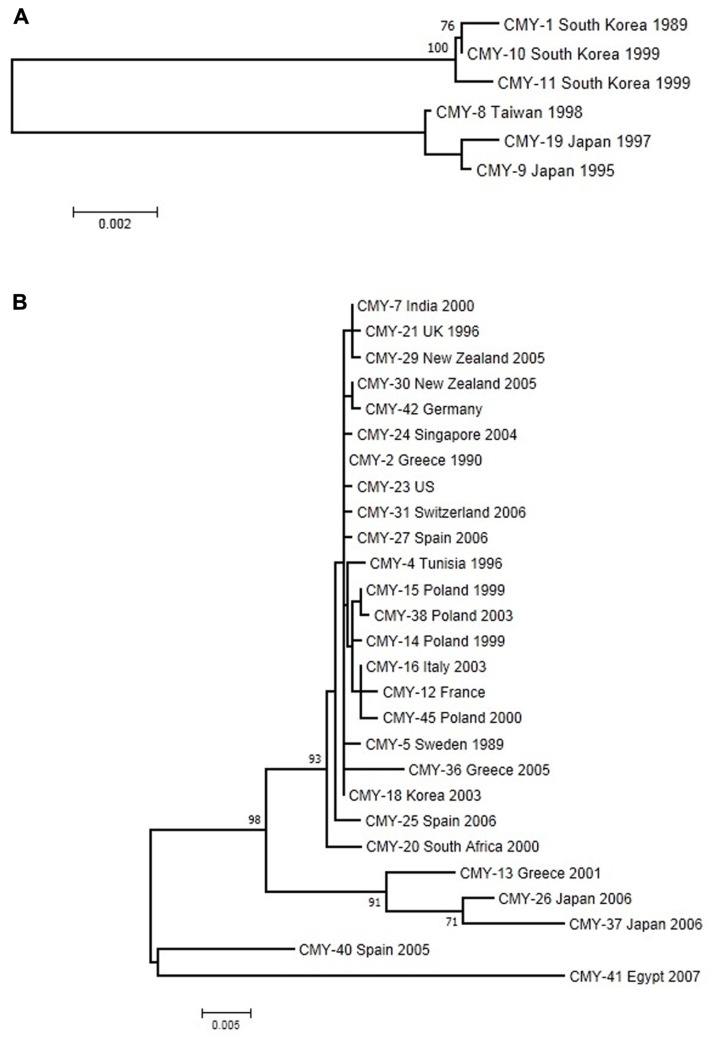
**Maximum-likelihood tree of CMY genes obtained with the Tamura-3P + G model of evolution**. **(A)** The upper tree corresponds to CMY-1 group. **(B)** The lower one to the CMY-2 group of sequences. Multiple sequence alignments were obtained using the corresponding amino acid sequences with Muscle (Edgar, 2004a,b). Maximum-likelihood trees were obtained using the most appropriate model of evolution resulting from the comparison of Bayesian Information Criterion (BIC) values for 24 alternative models including gamma-distributed heterogeneous rates of evolution among and invariant sites. Support of the inferred clusters was evaluated with 1000 bootstrap replicates, BS values >70% are indicated. All these methods were used as implemented in MEGA 5 (Tamura et al., 2011). The scale bar corresponds to substitutions/site. Detection of positive selection and neutrality tests were performed as described in **Figure 2**.

Plasmid-encoded *ampC* genes have been known since 1989 when CMY-1 was described in *K. pneumoniae* isolates (Bauernfeind et al., 1989), while the most common plasmid-mediated AmpC β-lactamase worldwide is CMY-2 (Jacoby, 2009). Generally, CMY-2 enzymes are responsible for outbreaks across European countries (D’Andrea et al., 2011). CMY-1 and CMY-2 have different phylogenetic origins (**Figure 3A**); whereas CMY-1 and related variants are close to chromosomally determined AmpC enzymes in Aeromonadaceae, CMY-2 and evolved variants are related to AmpC from *Citrobacter freundii* (Barlow and Hall, 2002a). A total of 32 CMY alleles with known date and country of first isolation were included in this analysis. These variants cluster into two well-defined and distant groups in the reconstructed ML tree using the Tamura-3P + gamma model (**Figure 3**), denoted CMY-1 and CMY-2. The CMY-2 group is larger and more diverse. A large number of sequences (*n* = 22) from the five continents are very similar and have diversified very recently, while the five remaining sequences in the CMY-2 group have a more ancient origin. This larger group is dominated by isolates from *E. coli*, but a recent colonization and spread into *Proteus mirabilis* can be observed in sequences isolated from European countries (D’Andrea et al., 2011) with a possible origin in North Africa (CMY-4 from Tunisia). The CMY-1 group included only six sequences from Asian–Pacific countries, which are distributed evenly in two subgroups of very similar sequences.

The CMY alignment resulted in 376 codons available for selection analysis. None of them presented significant deviations from the expected dN-dS value under neutrality, although 123 codons had positive values for this difference. These analyses corresponded to the whole data set of CMY alleles considered but, as shown above, there are two main, anciently-diverged groups that have been analyzed separately in search of positive selection. The phylogeny-based analysis of CMY-1 variants failed to identify any codon with a significant positive difference between dN and dS, with only seven codons yielding positive values of this parameter none of which reached statistical significance. Similar results were obtained for the CMY-2 group, with average dN–dS values of -0.362 (range: -10.484 to 1.000, minimum *P* = 0.444), and the two pairwise comparison-based tests failing to reveal evidence for positive selection. Barlow and Hall (2002a) obtained similar results using a lower number of variants. Although a diversification process has occurred in this group, especially in CMY-2, the newly arisen variants were not subject to positive selection, in contrast to TEM and CTX-M enzymes. The hydrolytic activities conferred by chromosomal *ampC* recovered from the pre-antibiotic era are essentially the same as plasmid-mediated *ampC* alleles, probably because they are all resistant to many β-lactam antibiotics. Therefore, the plasmid-mediated AmpC enzymes are not evolving phenotypically because it is not necessary (Barlow and Hall, 2002a). However, the impact of single-amino acid substitutions on the evolution of CMY proteins can be observed in particular cases. For instance, the G214E change in CMY-2 increases the catalytic efficiency against cefotaxime (Endimiani et al., 2010), suggesting that although the selective pressure occurs it might be weak.

β-Lactamases with capacity for hydrolyzing carbapenems are very versatile in their origins and nucleotide sequence. Currently, almost 30 families of carbapenemases belonging to classes A, D, and B are known (Widmann et al., 2012), but new families are being described continuously (Pollini et al., 2012). The spread of metalo-β-lactamases (MBL), classified as class B, presents a major challenge both for treatment of individual patients and for policies of infection control (Cornaglia et al., 2011), because they confer resistance to almost all β-lactams, except aztreonam. There are at least nine different types of MBL, but probably IMP and VIM are the most prevalent (Fukigai et al., 2007; Van der Bij et al., 2011) and diversified ^1^. The dendrogram of VIM enzymes suggests two recent events of diversification involving VIM-1 and VIM-2 (Cornaglia et al., 2011).

**FIGURE 4:**
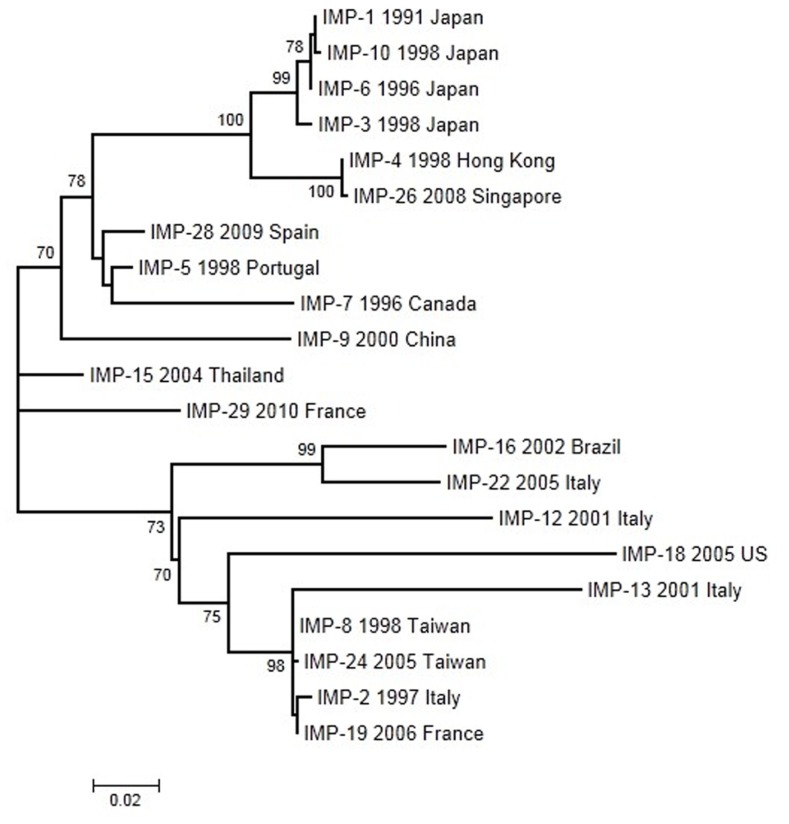
**Maximum-likelihood tree of IMP genes obtained with the Tamura-3P model of evolution**. Multiple sequence alignments were obtained using the corresponding amino acid sequences with Muscle (Edgar, 2004a,b). Maximum-likelihood trees were obtained using the most appropriate model of evolution resulting from the comparison of Bayesian Information Criterion (BIC) values for 24 alternative models including gamma-distributed heterogeneous rates of evolution among and invariant sites. Support of the inferred clusters was evaluated with 1000 bootstrap replicates, BS values >70% are indicated. All these methods were used as implemented in MEGA 5 (Tamura et al., 2011). The scale bar corresponds to substitutions/site. Detection of positive selection and neutrality tests were performed as described in **Figure 2**.

We have analyzed 21 IMP alleles with year and country of first isolation. The ML tree was constructed using the Tamura-3P model. According to this phylogeny (**Figure 4**), several closely related groups of likely recent origin and many other more anciently diverged sequences can be observed. The two recently diverged groups include mostly sequences isolated from East Asian countries. A common feature of these recently spread groups is that they include most alleles isolated in species not belonging to *P. aeruginosa* and *A. baumannii*. The first IMP allele, IMP-1, was isolated in Japan and it is included in a group with three additional alleles from the same country. Another group related to this one includes two sequences isolated in Hong Kong and Singapore, respectively. The second recently spread group includes two sequences from Taiwan and one from Italy and France. In both groups, the V67F change was selected on three occasions suggesting that this change could be under positive selection. However, no mutation presented a statistically significant, positive deviation in the dN–dS parameters indicative of positive selection acting in the IMP phylogeny. Pairwise comparisons also failed to reveal positive selection as a driving force in the evolution of these alleles. The V67F change is highly variable, non-essential for the protein function but it could have a role in antibiotic recognition (Widmann et al., 2012). Moreover, in the IMP-1 clade, the mutation G235S contributes in increasing the hydrolytic activity against meropenem (Liu et al., 2012), suggesting a process of selection.

Despite their many differences at the genetic and ecological levels, the three families of β-lactamases present several remarkable similarities. The three gene families have several anciently diverged variants along with others that have spread very recently. Most of these recently diverged groups include very similar sequences with only a few nucleotide substitutions and usually only one or two amino acid replacements. This distribution probably results from a biased screening of variants – only those with at least one amino acid difference are reported and deposited in the corresponding databases, which may lead to the false impression that positive selection is a major factor driving the evolution of these genes. In fact, our analyses in search of positive selection in these gene families failed to find evidence for it after using both phylogeny- and pairwise-based analysis of dN–dS differences in codons or the whole gene. In general, most isolates in recently spread OXA- and IMP-groups are derived from *A. baumannii* and *P. aeruginosa.* Over 210 and 120 β-lactamases have been identified in *A. baumannii* and *P. aeruginosa*, respectively, suggesting that these species are crucial reservoirs of β-lactam resistance determinants (Zhao and Hu, 2010, 2012). Although they are ubiquitous in nature also they are frequently isolated in nosocomial and chronic infections, exposed to a variety of antibiotic regimens, giving rise to selection and spread of mechanisms of resistance. In this scenario these results suggest that these species are an excellent shuttle of resistance genes between the environment and pathogenic strains.

## UNDERSTANDING THE ADAPTIVE POSSIBILITIES AND THEIR CONSTRICTIONS TO PREDICT β-LACTAMASE EVOLUTION

The ability of protein to evolve is dependent on tolerance to change. TEM β-lactamases can be extremely tolerant to amino acid substitutions both in the number of affected positions (>200 positions can change; Salverda et al., 2010) as in number of amino acids in a particular position (one-third of the positions in TEM can tolerate more than five different amino acids; Deng et al., 2012). The combination of potential mutations could increase the theoretical diversity up to astronomically large numbers. However, only 82 polymorphic positions have been described among the 204 TEM mutants described so far and, strikingly, 85% of the substitutions are located in only 17 positions.^1^ Moreover, the most polymorphic positions show only 3–4 amino acid changes. So, why does not the sequence plasticity observed in site-directed mutagenesis experiments translate easily in huge diversification of evolved proteins in nature? (Taverna and Goldstein, 2002).

Many researchers have studied the possible genetic constraints involved in the discrepancy between the potential and the actually observed biological plasticity of β-lactamases (Camps et al., 2007). Mutagenesis studies on TEM-1 did not yield TEM variants more efficient than TEM-1 in hydrolyzing natural β-lactams such as ampicillin and cephalosporin C, suggesting that during thousands of years of evolution TEM-1 has become the most efficient enzyme in conferring the highest MIC values to natural β-lactams (Deng et al., 2012). The kinetic effect of this *evolved enzyme* is the result of a delicate network of hydrogen bonds contributing to the enzymatic stabilization. Therefore, it is easy to imagine that any mutation could alter this balance and consequently decrease its efficiency. Internal positions (close to the active center) are less tolerant of substitutions than external ones because, presumably, they have more interactions and the equilibrium is altered easily (Deng et al., 2012). Therefore, the majority of mutations in internal positions are generally deleterious but they might also facilitate the acquisition of new functions (Tokuriki et al., 2008). For instance, mutations such as R164H in TEM-1 (TEM-29), D179N in SHV-1 (SHV-8), or P167T in CTX-M-1 (CTX-M-58) increase the hydrolytic activity against third generation β-lactam antibiotics (*new* function), but decrease the specific activity of the existing function as a consequence of a loss of stability (Poirel et al., 2001; Wang et al., 2002). The protein instability observed with some mutations leads to lower levels of correctly-folded protein and, consequently, to a reduction in enzymatic activity (Bloom et al., 2005). Therefore, the stability/instability level of a mutant protein must be proportional to its fitness. Almost 40% of TEM-1 artificial mutations cause a partial or complete reduction in the level of folded protein (Tokuriki and Tawfik, 2009). Hence as mutations accumulate, the likelihood of declines in protein fitness increases exponentially (Soskine and Tawfik, 2010).

According to the theoretical estimations, those proteins carrying, five mutations on average, with respect to the wild-type variant, will reduce their fitness in > 80% (Tokuriki and Tawfik, 2009) and the accumulation of >10 mutations per gene would result in non-functionalization of 99% of the mutated genes (Soskine and Tawfik, 2010). However, several TEM-1 variants, such as TEM-121, TEM-162, or TEM-194, carrying five or more mutations have been described in nature. The increased tolerance to changes observed in these enzymes requires the presence of mutations capable of compensating the loss of stability due to previous selection of mutations conferring *new* functions (Kather et al., 2008). In other words, the evolution toward *new* functions must be facilitated by mutations that act as compensatory mutations of protein stability defects (Brown et al., 2010).

The best studied example of compensatory mutation is M182T in TEM enzymes. This mutation has no effect on the catalytic activity of TEM-1 (Huang and Palzkill, 1997), however the thermodynamic stability of TEM-1 is increased by 2.67 kcal/mol in the presence of this mutation (Wang et al., 2002). Therefore, the selection of M182T will be favored when previous mutations that increase the activity against new β-lactams have reduced protein stability (Camps et al., 2007). For instance, TEM-15 has substitutions E104K:G238S which contribute synergistically to increase the hydrolytic activity toward extended-spectrum cephalosporins. These mutations rearrange the active site and, consequently, destabilize the enzyme (∆∆G = 2.24 kcal/mol), whereas the triple mutant, TEM-52, contains the additional substitution M182T compensating the loss associated with the E104K:G238S substitutions (∆∆G = -1.76 kcal/mol), increasing the activity against extended-spectrum β-lactams (Wang et al., 2002). Progressively, other compensatory mutations have been described in TEM enzymes (Brown et al., 2010).

The presence of compensatory mutations is a global phenomenon, also described in other β-lactamases, such as mutations A77V in CTX-M (Novais et al., 2008), N70S in metallo-β-lactamase BcII (Tomatis et al., 2008), or L169I in OXA and ROB-β-lactamases (Poirel et al., 2002; Galán et al., 2003). From an evolutionary perspective, these point mutations are essential because the *extra* stability promotes the evolvability of the protein (Bloom et al., 2006), but potentially they can also have negative effects if the excess stability hinders protein turnover (Brown et al., 2010). Therefore, an equilibrium between evolvability and robustness is essential for the plasticity of proteins, that is, between the necessity to adapt to new environments and the ability to maintain a phenotype in the presence of genotypic variations.

The network model establishes strong associations between particular mutations. For instance, mutation M182T is more frequently associated with mutations related to ESBL phenotypes (Huang and Palzkill, 1997), whereas N276D is associated with IRT phenotypes (Abriata et al., 2012), suggesting co-evolutionary processes (Guthrie et al., 2011). In other cases, mutation L210P is co-selected with R244A (Marciano et al., 2008), whereas mutation E240K is associated with R164H, suggesting different networks of compensatory mutations depending on the initial mutation responsible for the ESBL phenotype. These epistatic constrictions required for maintaining stability while activity is increased drastically reduce the number of possible mutational pathways (Weinreich et al., 2006; Novais et al., 2010). Therefore, initial mutations drive evolutionary pathways of protein evolution (Salverda et al., 2011). The genetic reconstruction based on a combinatorial strategy of five mutations in TEM-1, which were chosen for their large joint phenotypic effect, revealed that only 18 of 120 evolutionary trajectories were accessible through Darwinian selection (Weinreich et al., 2006). Our group, using the CTX-M enzyme as model, obtained similar results but concluded that only the simultaneous presence of cefotaxime and ceftazidime in the environment permitted reaching the highest level of resistance (Novais et al., 2010). These studies of experimental evolution on β-lactamases show that only a small fraction of all possible mutational trajectories are accessible to evolution.

These lines of evidence suggest that many combinations of mutations must be antagonistic. Pleiotropic antagonism has been used to describe the incompatibility between ESBL and inhibitor-resistant β-lactamases phenotypes (Cantón et al., 2008; Ripoll et al., 2011). However, in this case pleiotropic antagonism is defined as the incompatibility between mutations conferring the same phenotype. For instance, mutations D240G and P167S increase the hydrolytic activity of CTX-M enzymes to ceftazidime. However, the double mutant confers lower MIC values than both single mutants in every background (Novais et al., 2010). Similar results were observed between mutations R164S and G238S in TEM, which show a strong negative interaction (Salverda et al., 2011). Laboratory-directed protein evolution experiments revealed that P167S and G238S mutations were more frequently selected (Barlow and Hall, 2002c; Novais et al., 2008) which are known to cause the greatest increase in CAZ and CTX resistance among all known single CTX-M and TEM mutations. However, there are more clinical isolates carrying the D240G and R164S mutations in CTX-M and TEM, respectively. This observation is contradictory to the Darwinian paradigm of evolution, as genotypes with higher fitness must be favored by selection (“survival of the fitness”).

At high mutation rates or weak bottlenecks, selection favors genotypes with larger networks of interactions, which will be more tolerant (robust) to the impact of deleterious mutations in spite of lower fitness. This phenomenon is known as “survival of the flattest” (Codoñer et al., 2006; Archetti, 2009). Depending on the environmental conditions, selection may favor an organism that replicates faster (“survival of the fitness”) or is more robust (“survival of the flattest”), but not both at the same time (Codoñer et al., 2006). The high connectedness of robust genotypes will be a guarantee of innovation without drastically losing functionality in the long time scale (Ferrada and Wagner, 2008), whereas a high evolvability is a guarantee for survival to strong bottlenecks in a short time but at the cost of losing many interactions and *old* functions. In general, β-lactamases carrying D240G or R164S have accumulated more mutations and the *old* functions are less affected than in β-lactamases harboring P167S or G238S mutations in the first step. Therefore, D240G or R164S mutations in CTX-M and TEM enzymes will condition the “survival of the flattest” model; whereas P167S or G238S mutations will contribute to “survival of the fittest.” These results suggest that the first mutations not only determine the co-selection of specific compensatory mutations but also condition the selection of certain evolutionary strategies. Nevertheless, the first mutations to be selected will also depend on the frequency and intensity of changes in the environment.

Antibiotic resistance is a public health problem and for many microbiologists and evolutionary researchers a goal to address the resistance problem has been to predict the selection of antibiotic resistance to new antibiotic (Martínez et al., 2007). In this situation, which types of studies are necessary to analyze in more depth the development of resistance in human bacterial pathogens? For many years, studies based on serial-passages experiments have been used as the best model to predict the selection of mutations involved in extending the spectrum of enzymatic activity (Martínez et al., 2011). However, this approach generally detected single mutants and, as a consequence of the strong bottlenecks involved in this experimental design, in most cases only those fitness variants corresponding to the “survival of the fitness” concept were recovered (Novais et al., 2008; Ripoll et al., 2011). This simple vision does not predict the selection of complex mutants based on the “survival of the flattest” concept. Then, the evolvability of known antibiotic resistance genes was explored by DNA shuffling and error-prone PCR (Orencia et al., 2001). The combination of both approaches in fluctuating environments could be more realistic, because in these cases mutations representing the “survival of the flattest,” such as R164S, are easily selected (Blázquez et al., 2000; Barlow and Hall, 2002c). Two new approaches are contributing to improve our predictive capacity. One of them, defining the driving forces the selection in a given combination of time and environment and understanding the possible evolutionary trajectories in the diversification process (Weinreich et al., 2006; Novais et al., 2010). Secondly, the implementation of bioinformatics programs such as FoldX (Deng et al., 2012), determining the ∆∆G of a new mutant and consequently its stability and fitness. The combinatorial approaches between bioinformatics and *in vitro* procedures will give rise to a more complete vision on the complex process of antibiotic resistance evolution.

## Conflict of Interest Statement

The authors declare that the research was conducted in the absence of any commercial or financial relationships that could be construed as a potential conflict of interest.

## Footnotes

1 http://www.lahey.com
